# Management of anterior cruciate ligament revision in adults: the 2022 ESSKA consensus: part II—surgical strategy

**DOI:** 10.1007/s00167-023-07550-5

**Published:** 2023-09-12

**Authors:** Vincenzo Condello, Philippe Beaufilis, Roland Becker, Sufian S. Ahmad, Marco Bonomo, David Dejour, Karl Eriksson, Giuseppe Filardo, Matthias J. Feucht, Alberto Grassi, Adrian Wilson, Jacques Menetrey, Nicolas Pujol, Martin Rathcke, Romain Seil, Marc J. Strauss, Thomas Tischer

**Affiliations:** 1 Department of Orthopaedic and Trauma Surgery, Waldkrankenhaus Erlangen, Erlangen, Germany; 2 Department of Orthopaedic Surgery, University Medicine Rostock, Rostock, Germany; 3 ESSKA Consensus Projects Advisor, Versailles, France; 4 Centre of Orthopaedic and Traumatology, University of Brandenburg an der Havel, Brandenburg, Germany; 5 Department of Orthopaedic Surgery of the Medical School of Hannover MHH, Annastift Hospital, Hannover, Germany; 6 Dipartimento di Ortopedia e Traumatologia, IRCCS Ospedale Sacro Cuore don Calabria, Negrar, VR Italy; 7 Lyon Ortho Clinic, Clinique de la sauvegarde Ramsay Santé, 29 avenue des sources, Lyon, France; 8 Department of Orthopaedics, Stockholm South Hospital, Karolinska Institutet, Stockholm, Sweden; 9 Applied and Translational Research Center, IRCCS Istituto Ortopedico Rizzoli, Bologna, Italy; 10 Faculty of Biomedical Sciences, Università della Svizzera Italiana, Lugano, Switzerland; 11 Department of Orthopaedic Surgery Paulinenhilfe, Diakonie Klinikum, Stuttgart, Germany; 12 Department of Orthopaedics and Trauma Surgery, Medical Center, Faculty of Medicine, Albert-Ludwigs-University of Freiburg, Freiburg, Germany; 13 II Clinica Ortopedica e Traumatologica, IRCCS Istituto Ortopedico Rizzoli, Bologna, Italy; 14 Orthopaedic Specialist Group, Harley Street Specialist Hospital, Queen Anne St, London, UK; 15 Center of Sports Medicine and Exercise, Hirslanden Clinique La Colline, Geneva, Switzerland; 16 Orthopaedic Surgery Service, University Hospital of Geneva, Geneva, Switzerland; 17 Centre Hospitalier de Versailles, Department of Orthopaedic and Trauma Surgery, Le Chesnay, France; 18 Department of Orthopaedics and Sportstraumatology, Bispebjerg-Frederiksberg Hospital, University of Copenhagen, Copenhagen, Denmark; 19 Department of Orthopaedic Surgery, Centre Hospitalier – Clinique d’Eich and Luxembourg Institute of Health, Luxembourg, Luxembourg; 20 Idrettens Helsesenter, Oslo, Norway; 21 Joint Preservation and Reconstructive Surgery and Sports Medicine Unit, Humanitas Castelli Clinic, Bergamo, Italy; 22 Department of Orthopaedic Surgery, Clinica San Francesco - GHC Group, Verona, Italy

**Keywords:** Anterior cruciate ligament, Revision, Failure, Surgery, Consensus, Surgical strategy

## Abstract

**Purpose:**

The aim of this ESSKA consensus is to give recommendations based on scientific evidence and expert opinion to improve the diagnosis, preoperative planning, indication and surgical strategy in Anterior Cruciate Ligament revision.

**Methods:**

Part 2, presented herein, followed exactly the same methodology as Part 1: the so-called ESSKA formal consensus derived from the Delphi method. Eighteen questions were ultimately asked. The quality of the answers received the following grades of recommendation: Grade A (high level scientific support), Grade B (scientific presumption), Grade C (low level scientific support) or Grade D (expert opinion). All answers were scored from 1 to 9 by the raters. Once a general consensus had been reached between the steering and rating groups, the question–answer sets were submitted to the peer-review group. A final combined meeting of all the members of the consensus was then held to ratify the document.

**Results:**

The review of the literature revealed a rather low scientific quality of studies examining the surgical strategy in cases of ACL reconstruction failure. Of the 18 questions, only 1 received a Grade A rating; 5, a Grade B rating; and 9, grades of C or D. The three remaining complex questions received further evaluations for each portion of the question and were looked at in more detail for the following grades: B and D; A, C and D; or A, B, C and D. The mean rating of all questions by the rating group was 8.0 + − 1.1. The questions and recommendations are listed in the article.

**Conclusion:**

ACL revision surgery, especially the surgical strategy, is a widely debated subject with many different opinions and techniques. The literature reveals a poor level of standardization. Therefore, this international European consensus project is of great importance and clinical relevance for guiding the management of ACL revision in adults.

**Level of evidence:**

Level II.

## Introduction

Anterior cruciate ligament (ACL) revision (ACLRev) is more demanding than primary reconstruction, and the outcomes are worse [9, 27]. Therefore, special emphasis must be placed on using the appropriate diagnostic tools for preoperative planning and surgical strategy and defining the best indications for a revision procedure [19]. As a result of the challenging aspects of ACLRev surgery, a European consensus was established to standardize the diagnosis, preoperative planning, surgical strategy and indications for ACLRev procedures. While the diagnostics and preoperative planning have already been published (Part 1) [18], the aim of this paper is to address in detail the surgical strategy (Part 2).

Surgical strategy plays a key role in performing optimal ACLRev, correcting previous technical errors and addressing associated pathologies. Multiple factors must be considered to optimize the outcome. Range of motion deficits or preexisting hyperextension should be taken into account. Choosing the best graft is a further problem, as it can be limited in the revision situation, and the surgeon must be knowledgeable in multiple graft options and preparation techniques. Previous tunnel widening may limit the possibility of performing a one-stage revision, and multiple strategies exist in the management of widened and misplaced tunnels [26]. One further consideration in the revision situation is previous graft fixation and hardware issues.

More recently, focus has been placed on limb alignment, which contributes to ligament and cartilage loading and must be considered in revision cases, both in the coronal and sagittal planes [12, 20]. It is well recognized that joint surface cartilage damage is more common in revision surgery and negatively influences the outcome [4]. Therefore, osteotomy performed in the coronal plane to unload either the medial compartment, which is the more frequently damaged, or the lateral side of the knee, is a key consideration in the management of the failed ACL and subsequent revision procedure [20]. Meniscal status has also been given more attention in recent years and is increasingly recognized as being key to the successful outcome of a revision procedure [14]. The debate on how to manage root, and especially ramp, lesions is ongoing [7]. Concomitant ligament insufficiency negatively influences the outcome of revision ACL surgery as well, and the current debate is especially focused on the management of associated medial-sided injuries [3, 25]. The amount of laxity also influences the surgical strategy, and the discussion of adding extraarticular lateral stabilization or carrying out slope-changing osteotomy are important factors to be considered [1, 10]. Bone quality may influence the choice of graft fixation. Finally, patient activity and expectations are of increasing importance for the indication of ACLRev surgery [8].

The aim of this European Society for Sports Traumatology, Knee Surgery and Arthroscopy (ESSKA) Consensus is to provide a combination of “scientific” evidence-based and expert-based recommendations regarding the diagnosis, preoperative assessment, indication and management of patients with failed ACL reconstruction to further improve the outcome after ACLRev [18, 19]. In this second part of the ACLRev consensus, the focus is on surgical strategy, with insight into the scientific evidence and challenges in the field, as well as clinical relevance for guiding treatment in clinical practice. The entire project can also be read in more detail on the ESSKA website (http://www.esska.org/page/projects). The reader is cautioned that this is not a complete systematic literature review and, further, that this consensus is not focused on any specific surgical technique.

## Material and methods

A European ACLRev consensus project was established by the European Society of Sports Traumatology, Knee Surgery and Arthroscopy (ESSKA) between 2020 and 2022, focusing on the management of primary ACL reconstruction failure in adults. ACL revision was defined as “all surgical procedures involving replacement of the ACL graft with a new graft”. Exclusion criteria for the consensus were patients under 18 years of age, multiligament injuries involving the ACL and PCL, and any procedure where the ACL was not revised and where any additional graft was inserted. Patients who had already undergone their first revision ACL procedure were excluded.

In this consensus, knee instability and laxity are defined as follows: pathological laxity as a clinical sign is “an increased passive response of a joint to an externally applied force or torque in biomechanical terms”. Laxity tests are carried out to assess knee injury and are used to evaluate the passive limits of motion in a particular direction or plane. In addition, instability is defined as a functional symptom where “an abnormal dynamic joint motion occurs in response to the complex, high-magnitude loads encountered during activities of daily living and sport activities.” For the purpose of this consensus, partial meniscectomy has been defined as resection of up to 50% of the meniscus depth with intact roots and no meniscus extrusion.

The process of this consensus project was similar to previously published ESSKA consensus projects [6, 13] and known as Formal Consensus as described by the French National Health care Institution (Haute Autorité de Santé HAS [11]). The details of this consensus are already published in Part I [18], so this will be only briefly described here. At the end of a thorough literature review and discussion, 18 questions focusing on surgical strategy were formulated and answered by the steering group. The quality of the answers was graded based on the quality of the available studies and was sorted into the appropriate grade of recommendation [19]. Grade A was defined as a high level of scientific support, Grade B as a scientific presumption, Grade C as a low level of scientific support, and Grade D as an expert opinion. After a general agreement was achieved within the steering group, the question–answer set was then submitted to the rating group, which consisted of 19 European specialist orthopaedic knee surgeons. Each member of the rating group was asked to score the question–answer sets according to the scientific evidence and their clinical experience using the Likert scale, which ranges from 1 (totally inappropriate) to 9 (totally appropriate). Suggestions from the participants were included after the first round. A revised draft was then prepared and resubmitted to the rating group for a second assessment. Then, the combined steering and rating groups had a meeting to finalize the draft document. The question–answer sets were then sent to the national societies affiliated with ESSKA. Fifty-one surgeons from 32 societies replied. The purpose of this unbiased peer-review group was to evaluate the question–answer set of the manuscript after grading by the rating group to determine the relevance, accuracy, geographic adaptability and clarity of the proposed recommendations. To conclude the consensus project, a final meeting between the steering and literature groups took place.

## Results

After the second round, recommendations were rated with an average of 8.0 + − 1.1 (out of nine). The mean points assigned by each rater ranged from 7.7 to 8.7 points. Five of the 18 answers received a score of less than 8 points, and all others were scored above 8.0 points. All the answers were thus considered appropriate. Only one question was rated as Grade A, while two further questions were rated as Grade A for only a part of that question; five questions and a component of two further questions were rated as Grade B, indicating that only a low-to-moderate level of scientific evidence is available for most of the answers. A very similar finding was made for the first part of this consensus study.

### Questions and answers

#### S1: Which factors are relevant to the surgical strategy when the decision is made to revise a previously reconstructed ACL?

The following factors are relevant to the surgical strategy:

**Table:** 

Range of motion	Severely restricted ROM, significant hyperextension (> 5°)
Availability of graft material	Autograft or allograft? Ipsilateral or contralateral graft harvesting? Bone block or soft tissue graft?
Previous tunnel size and location (Fig. 1)	Are the tunnel diameters of preexisting tunnels acceptable? Can the tunnels be reused or are new tunnels necessary? Can new tunnels be drilled without creating a bony defect (confluent tunnels)? Can stable fixation be achieved?
Previous graft fixation	Is it necessary to remove previous fixation material? Will removal of fixation material create a relevant bony defect?
Limb alignment (coronal/sagittal)	Is limb alignment a possible factor for ACL graft failure? Can limb alignment be corrected in a single-stage procedure or is a two-stage procedure preferred?
Meniscal status	Does a specific meniscal tear need to be addressed (root tear, ramp lesion)? Is significant meniscal loss a possible reason for ACL graft failure? Is meniscal reconstruction or transplantation necessary?
Cartilage status/preexisting OA	Is a cartilage repair procedure indicated? May an osteotomy to unload unicompartmental OA be an option?
Concomitant ligament insufficiency	Are there relevant concomitant ligament insufficiencies contributing to ACL graft failure? Can all ligaments be treated in a single-stage procedure? May the patient benefit from additional anterolateral stabilization?
Grade of laxity	Is concomitant anterolateral stabilization indicated? Is a posterolateral root tear or posteromedial ramp lesion present?
Bone quality	Can adequate fixation stability be achieved with standard fixation methods? Are alternative techniques necessary (e.g., back-up fixation or oversized screws?)
Patient activity and expectation	May the patient benefit from an additional anterolateral stabilization?
Infection status	Is an active infection evident? Suspected low-grade infection?

**Fig. 1:**
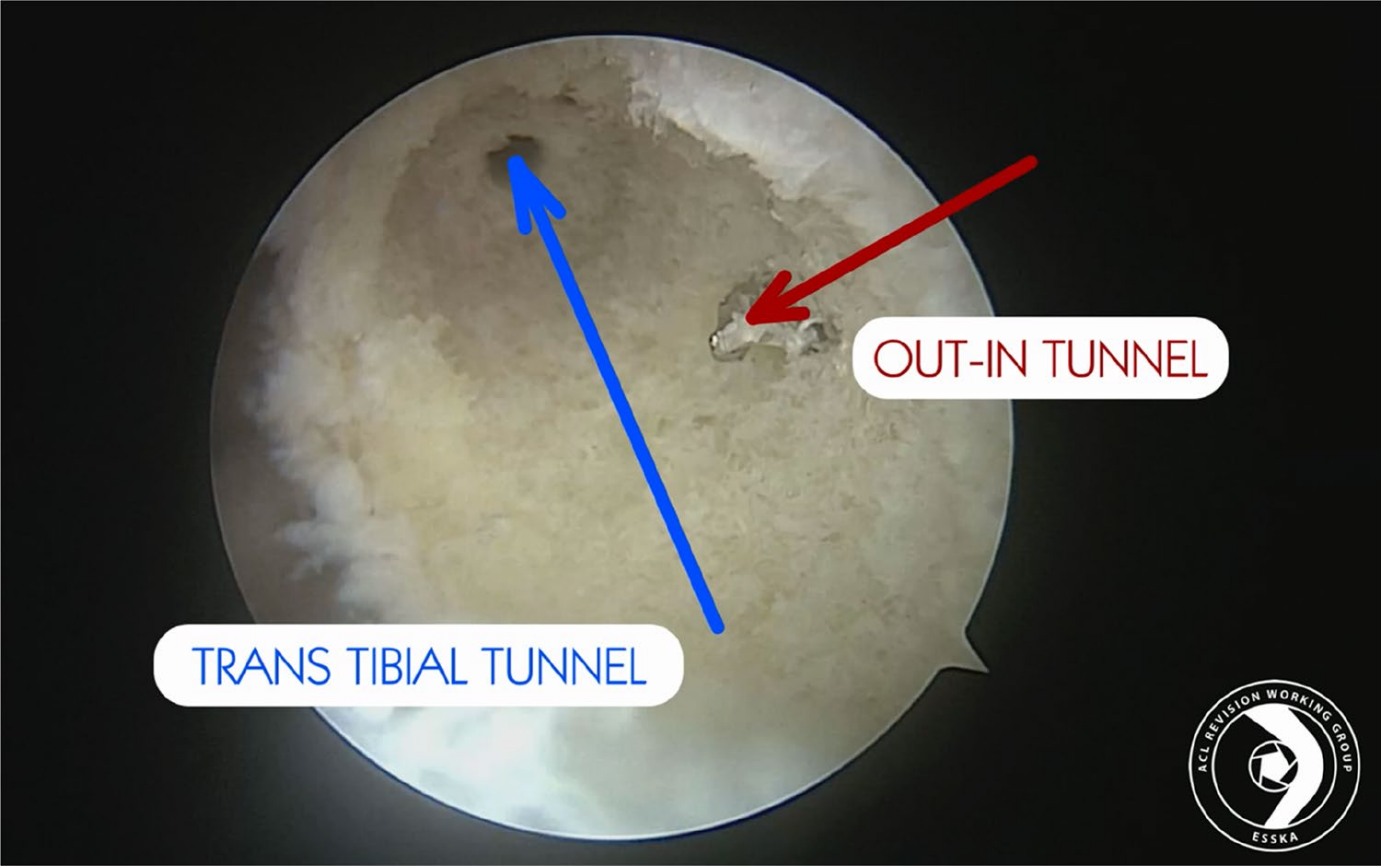
Example of a one-step surgical revision in which the new femoral tunnel (out-in red arrow) does only minimally interfere with the previous one (transtibial blue arrow)

#### S2: Which factors influence the decision to perform a single- versus two-stage procedure?

The following factors influence the decision of single- versus two-stage procedures:

**Table:** 

Absolute indications for a two-stage procedure	Relative indications for a two-stage procedure
Clinically relevant reduced range of motion due to arthrofibrosis	Tunnel widening around > 12 mm (depending on the graft choice, drilling technique, and fixation technique)
Infection/suspicion of infection	Partially malpositioned tunnel interfering with a new, anatomically placed tunnel
Impossibility of achieving secure graft fixation at the anatomic insertion sites due to insufficient bone stock	Complex combined surgery

#### S3: When is bone grafting of a widened or malpositioned tunnel indicated?

Bone grafting is generally recommended if secure graft fixation cannot be achieved in an anatomic position due to an increased tunnel diameter. No absolute threshold exists for the “critical tunnel diameter”, with values ranging between 12 and 15 mm. In fact, the threshold may vary with regard to graft choice, drilling technique, fixation technique, and knee size.

Three scenarios exist in which bone grafting may be indicated:

1. A previously partially malpositioned tunnel, which will interfere with a new anatomic tunnel, resulting in a confluent tunnel exceeding the critical diameter
2. A previous anatomic tunnel position exceeding the critical diameter
3. Intra-OP widening caused by difficult fixation hardware removal

However, by using specific techniques such as outside-in drilling with a different tunnel trajectory, over the top technique, using grafts with large bone blocks and large interference screws, bone grafting may not be necessary.

Bone grafting is usually performed as a two-stage procedure; however, with specific techniques (e.g., impaction bone grafting), bone grafting can also be performed as a one-stage procedure.

If preexisting tunnels do not interfere with new tunnel placement or graft fixation, they can usually be left alone, and bone grafting is not necessarily indicated.

#### S4: What is the best material for tunnel grafting (autograft, allograft, or synthetic bone substitutes)?

Both autologous and allogenic bone are suitable for tunnel grafting. Autologous bone is osteogenic, osteoinductive and osteoconductive, whereas allogenic bone is mainly osteoconductive. Therefore, autologous bone may represent the best graft material, but there is some donor side morbidity. Good filling rates have been reported with both graft materials [17]. Because of limited data and unfavorable results observed after open wedge high tibial osteotomy, synthetic bone substitutes should be used with care. Nevertheless, two studies have reported comparable results between autologous bone and silicate-substituted calcium phosphate [21, 22]. Whatever material is used, careful tunnel preparation with removal of all graft material and sutures and breaking up of sclerotic bone is important.

#### S5: When is it safe to perform ACLRev after staged bone grafting (time)?

An interval of 3–6 months of follow-up before second-stage revision, ACLRev is recommended after staged bone grafting, and CT imaging could be considered to determine adequate graft incorporation. Incorporation of allogenic bone may require a longer time period as compared to autologous bone.

#### S6: When is an additional osteotomy indicated to correct coronal malalignment (Varus/Valgus) in ACL revision surgery?

An osteotomy to correct coronal malalignment is indicated in patients with varus or valgus deviation ≥ 5° accompanied by early OA, significant cartilage damage and/or symptomatic meniscal deficiency and in patients with varus or valgus deviation associated with ligamentous insufficiency (e.g., a thrust phenomenon-dynamic joint space opening). The threshold of 5° is based on common indications for varus or valgus correction reported in the literature; however, a shift towards even smaller thresholds has occurred in recent years. Therefore, an osteotomy to correct varus or valgus deviation < 5° may be indicated in selected cases, such as patients undergoing concomitant meniscal transplantation, cartilage repair procedures, or collateral ligament reconstruction. An isolated varus malalignment without the abovementioned associated conditions is not an indication per se for an osteotomy.

#### S7: When is an additional osteotomy indicated to correct sagittal malalignment (slope) in ACL revision surgery?

**Fig. 2:**
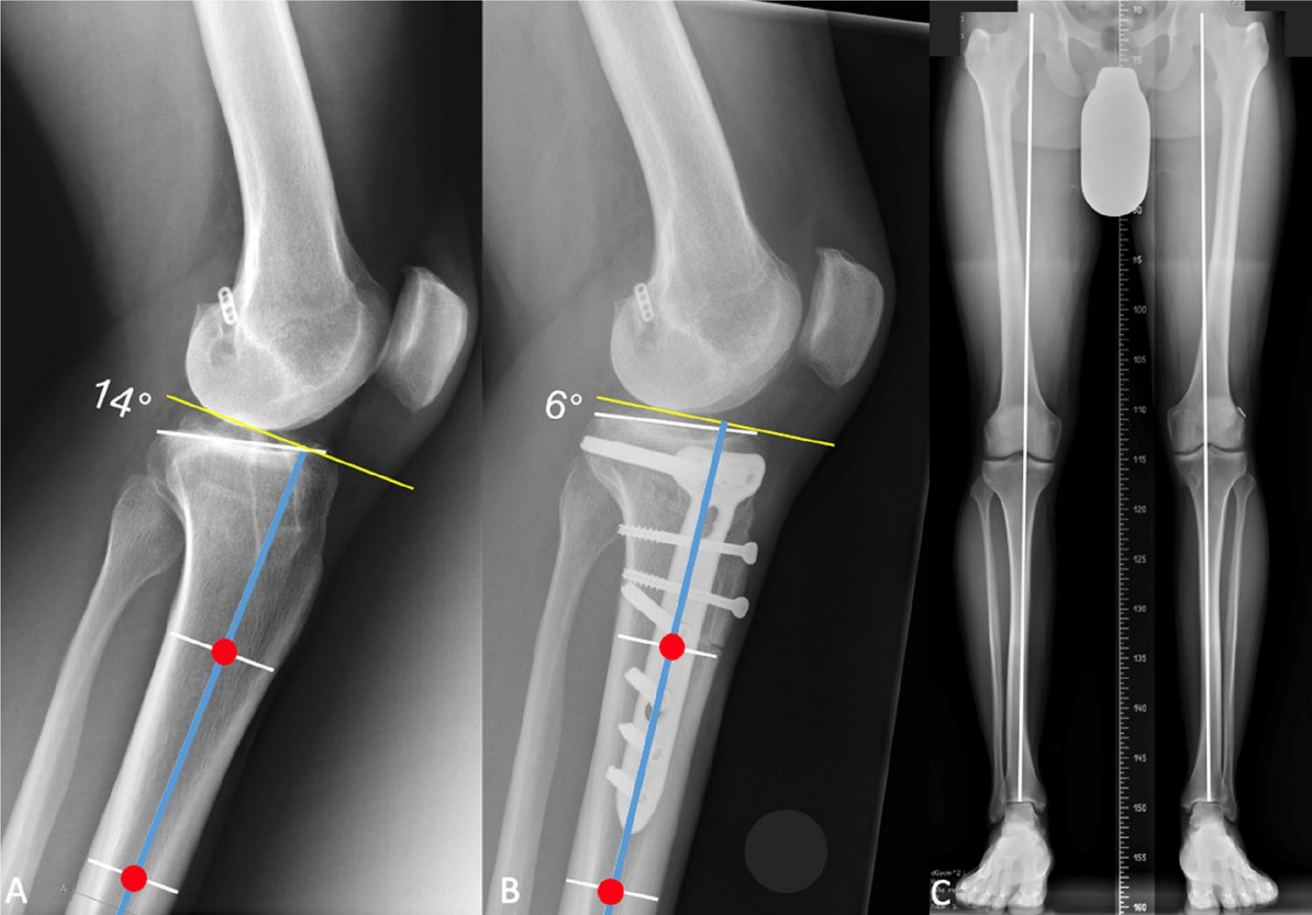
Increased posterior tibial slope (PTS) measured on lateral radiograph in a well-aligned patient with ACL re-rupture. The PTS is measured on a strictly lateral radiograph as follows: angle between the tangent of the anterior and posterior margin of the medial tibial plateau (white line) and the perpendicular to the longitudinal, mechanical axis (blue line) which is defined as the connection of two points (red dots) equidistant between the anterior and posterior border of the tibia at 5 and 15 cm below the tibial plateau. The post op X-ray shows a slope reduction of 8° after a transtuberosity anterior closing wedge osteotomy fixed with a plate plus two screws for tibial tubercle fixation

A slope-reducing osteotomy (extension osteotomy) should be considered in patients with failed primary ACL reconstruction and a PTS (posterior tibial slope) ≥ 12°, as measured on lateral radiographs (Fig. 2). The indication may be even stronger in patients with increased static anterior tibial translation (> 5 mm on a monopodal stance), multiple failed ACL reconstructions, and/or deficiency of the posterior medial meniscal horn. Careful consideration should be given if there is preexisting hyperextension of the knee, as this may be a contraindication.

#### S8: When is an additional extraarticular anterolateral procedure indicated in ACLR surgery?

Systematic use of additional extraarticular anterolateral procedure should be considered in revision ACL reconstruction, especially when patients present with gross laxity (pivot shift +++, Grades II and III [IKDC] of AP instability and/or in pivoting sports or in hyperlaxity). Additionally, check for laxity on the medial side, because it can also increase anterolateral instability. However, there is still a lack of high levels of evidence in existing studies.

#### S9: When should additional medial laxity be treated or addressed?

**Fig. 3:**
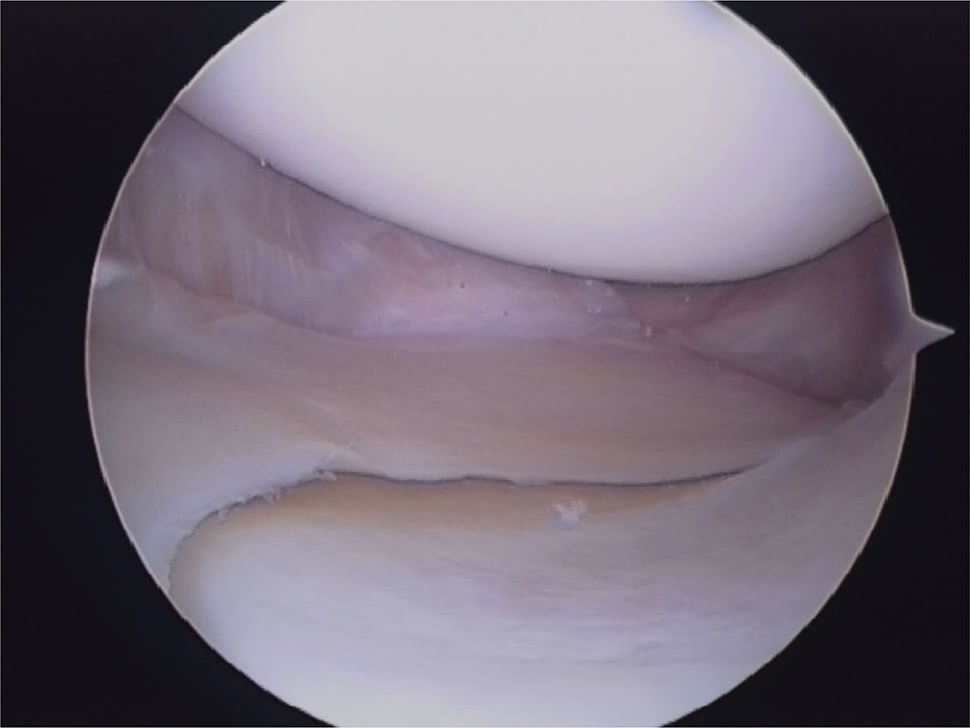
Positive drive through sign of the medial compartment in a patient with ACL and MCL rupture

Preoperative medial laxity is a risk factor for poorer ACL revision outcomes. Consideration should be given to concomitant medial collateral ligament (MCL) reconstruction for Grades 2 and 3 (IKDC C and D) MCL laxity (Fig. 3). However, high-quality comparative studies are lacking.

#### S10: When should additional lateral laxity be treated?

A true lateral laxity, including a subtle isolated fibular collateral ligament (FCL), posterolateral corner or complete lateral injury, should also be detected and is evidently associated with failure of revision. Clinical thresholds regarding gapping are lacking. However, a lateral or posterolateral injury must be clearly delineated from anterolateral instability (which does not cause gapping) and should be treated accordingly to prevent failure of the ACL revision graft.

#### S11: When could an additional meniscal substitute or meniscal allograft be indicated?

Meniscus substitutes and meniscus allografts are able to improve clinical outcomes in cases with selected indications. A chondroprotective effect is expected but not yet proven to occur over a long-term follow-up.

Substitute: With failed previous partial meniscectomy and meniscus-related complaints, an additional meniscal substitute may be considered in rare cases, in conjunction with ACL revision for such patients (Grade C). However, the implantation of a substitute at the same time as a partial meniscectomy (“prophylactic” substitute) is not recommended (Grade A).

Meniscus allograft: An additional meniscus allograft may be considered in conjunction with ACL revision in patients with failed previous total or subtotal meniscectomy and meniscus-related complaints without significant cartilage wear (Grade B). Meniscal allografting as a concomitant procedure in ACL revision reconstruction may be performed to aid in joint stability when meniscus deficiency is believed to be a contributing factor to failure (Grade D).

#### S12: Which factors influence the decision in graft choice for ACLRev?

Before choosing the proper graft in ACLRev, the following questions should be answered:

1. Which previous graft has been harvested?
2. Is there a need to fill bone tunnel(s)?
3. Is there a need for multiligament reconstruction?
4. What are the advantages and disadvantages of the different autografts (hamstring vs. quad vs. BPTB, …)?
5. What are the respective advantages and disadvantages of autografts versus allografts (see question 13). What is the allograft availability?
6. Is it pertinent to reharvest the same graft on the ipsilateral knee or harvest from the contralateral knee (see question 15)?
7. Are there abnormalities (e.g., degenerative changes or changes in the patellar height) of the patellofemoral joint?

Depending on these factors, the choice is often a compromise—in other words, a necessity rather than a real choice.

#### S13: Are allografts comparable to autografts regarding outcome?

Allografts are more frequently used in ACL revisions than in primary ACL reconstruction. Allografts offer the advantages of decreased operative times and low average pain during the entire rehabilitation period. The disadvantages of allografts include a risk of disease transmission, immune rejection, delay in remodelling, prolonged integration process and possibly higher revision rates, depending on graft processing. Historically, the results have been recognized to be inferior when using irradiated grafts. Nonirradiated grafts (cryopreserved or fresh frozen) are plausible alternatives to autografts, but it is unclear whether the failure rates are comparable, and caution still remains for the use of allografts in younger patients. Such patients tend to be more active, and there is increasing understanding of the higher risks in this age group. The evidence is weak, but allografts in young patients are likely to carry an increased risk of failure [9]. This is an area for further comparative work. The choice for using allografts is based on preference, while taking into account the longer maturation of allografts. Graft availability and donor-site morbidity become the dominant factors in decision-making in these clinical situations. Cost issues have also been underlined.

#### S14: Is there a role for synthetic grafts or synthetic augmentation?

The use of synthetic grafts is not recommended (Grade B). For synthetic augmentation, there are currently insufficient data for an evidence-based recommendation (Grade D).

#### S15: What is the place of graft harvesting from the contralateral knee and graft reharvesting from the ipsilateral knee?

Contralateral graft harvesting is considered a valid alternative to ipsilateral autografts or allografts.

Although ACL reconstruction using a reharvested BPTB tendon may be possible, the tendon quality (histologically) is lower than that from a primary tendon harvest, and there is no available literature concerning the quadriceps tendon.

#### S16: What is the minimal tendinous graft diameter in ACLRev surgery?

The same requirements regarding graft thickness and length are necessary for the grafts used in revision surgery as for those used in primary ACL surgery. A minimum graft diameter of 8 mm is advised and is dependent on many factors (knee size, surgical technique, type of graft,).

#### S17: What is the best treatment in the case of a planned ACLRev in a patient with a suspected low-grade infection?

Infection after primary ACL reconstruction has a low incidence, and there is little evidence to guide the correct treatment. However, in the case of a suspected infection, it is mandatory to perform blood tests and joint aspiration (white blood cell count, C-reactive protein, erythrocyte sedimentation rate, culture and microscopic examination). Tissue biopsy may be important to rule out low-grade infection in case of doubt and to identify bacteria (see also question D17).

A multistaged procedure in suspected low-grade infection is mandatory, with thorough debridement (including ACL graft remnant excision and hardware removal) and antibiotic therapy prior to second-stage ACL reconstruction.

#### S18: Is antibiotic soaking of grafts useful for reducing postoperative infections?

Soaking the graft in an antibiotic [vancomycin (Grade A), gentamycin (Grade C)] solution prior to graft implantation is a valid option to reduce the incidence of postoperative septic arthritis. However, the development of resistance may be of concern (Grade D).

**Table:** 

	Grade	Agreement
S1	B	8.6/9
S2	C	8.1/9
S3	C	7.8/9
S4	B	7.8/9
S5	C	8.0/9
S6	C	8.3/9
S7	B	7.7/9
S8	B	8.1/9
S9	B	7.4/9
S10	C	8.3/9
S11	A–D	7.7/9
S12	C	8.2/9
S13	A	8.2/9
S14	B/D	8.8/9
S15	C	8.1/9
S16	C	8.4/9
S17	D	8.0/9
S18	A/C/D	8.1/9

## Discussion

The results of this project have provided a consensus for ACLRev surgery. The conclusions are based on the input of experts from all over Europe. The proposed recommendations of the consensus are very important due to the limited general agreement in the management of failed ACL reconstruction. Of particular concern is to determine an appropriate surgical strategy tailored to the specific patient in question. Surprisingly, the ESSKA ACLR Consensus shows that a similar agreement regarding the surgical strategy has been reached, as was the case for the diagnostics and preoperative planning. The methodology of the process has already been discussed in Part I [18].

ACLRev is more demanding than primary reconstruction, and the outcomes are worse [9, 27]. Therefore, a meticulous diagnostic work-up is essential, and preoperative planning is of utmost importance for any surgical strategy [18]. While the diagnostic algorithm is quite clear, there are significant factors in the surgical strategy that are less clear. In particular, the exact thresholds for treating tunnel widening, the time to wait after bone grafting, amount of joint space opening or gapping, degree of tibial slope, etc. Such factors are difficult to map out and quantify. A consensus does not exist, for example, on tunnel widening, and this is reflected in the fact that some surgeons regard tunnels wider than 10 mm as difficult to manage in a single-stage procedure, while other experts always manage such tunnel widening in a single stage [15, 24, 26]. A threshold of greater than 12 mm was then defined as a relative contraindication for one-stage revision, but this depends on many factors, such as graft choice, drilling technique, fixation technique and surgical experience. This highlights the difficulties in defining exact thresholds. It is clear that the surgical strategy is fundamentally determined by preoperative diagnostics, surgical planning and the experience of the treating surgical team, as well as their ability to evaluate the best strategy and adapt general recommendations to any individual case.

Another difficult topic is when to perform an additional osteotomy, either to correct the tibial slope to improve stability or to unload a damaged compartment where there has been joint surface damage [20]. While the role of slope correction in the second revision now seems clear, this is still debated in first revision cases [1, 16, 23]. However, tibial slope correction and additional extraarticular anterolateral procedures can achieve good outcomes in difficult ACLRev cases [1].

Another difficult topic is when and how to address associated peripheral ligamentous lesions. Some surgeons are of the opinion that an additional extraarticular anterolateral procedure is key in every ACLRev case, whereas some other surgeons are more convinced that an individualized approach should be adopted. Factors that are in strong favour for an additional extraarticular anterolateral procedure are patients presenting with gross laxity (pivot shift +++, grade II and III (IKDC) of AP instability and/or in pivoting sports or in hyperlaxity).

The management of associated ligamentous lesions on the medial side is a much more controversial subject. Biomechanical studies have shown that partial and complete medial collateral ligament tears increase the load on the ACL [5]. This has also been shown in clinical studies that have demonstrated that MCL insufficiency (grade II and III) leads to higher failure rates of ACLRev [3]. Addressing MCL insufficiency during revision improves outcomes, which has been reflected in the literature by Alm et al. [2].

While the isolated effect of most procedures is often quite clear, complex combined surgeries are less well understood. Is tibial slope correction appropriate in the first ACLRev? Does this change according to meniscal status? Can some degrees of increased slope be addressed with additional extraarticular anterolateral procedures? These complex topics are currently evolving and may necessitate an updated consensus in the future.

There are some limitations in this work: the main one is that the scientific level of the answers is not homogeneous. Concerning the answers with a low level of scientific evidence, the opinion of the experts has been used to validate the proposed recommendations. In the future, research will probably provide us with different answers from those published in this article.

Since the level of scientific evidence is different for every question, a grading of recommendation was performed for each statement (Grade A–D).

More information about this consensus as well as about the included references can be found on the ESSKA website (http://www.esska.org/page/projects).

## Conclusion

The second part of the European consensus on ACLR surgery focuses on the most important aspects to be faced to plan an adequate surgical strategy after primary ACL reconstruction failure.

This represents a second step following the previously described work on diagnosis and preop planning. The consensus aims to propose a method for the standardization of the strategy for ACLRev surgery, according to current scientific evidence and clinical expertise. The ultimate goal is to improve the outcomes of this complex surgery, which requires surgical experience.
